# Patent landscape review of non-invasive medical sensors for continuous monitoring of blood pressure and their validation in critical care practice

**DOI:** 10.3389/fmed.2023.1138051

**Published:** 2023-07-11

**Authors:** Olena Litvinova, Aylin Bilir, Emil D. Parvanov, Josef Niebauer, Maria Kletecka-Pulker, Oliver Kimberger, Atanas G. Atanasov, Harald Willschke

**Affiliations:** ^1^National University of Pharmacy of the Ministry of Health of Ukraine, Kharkiv, Ukraine; ^2^Ludwig Boltzmann Institute Digital Health and Patient Safety, Medical University of Vienna, Vienna, Austria; ^3^Department of Translational Stem Cell Biology, Research Institute of the Medical University of Varna, Varna, Bulgaria; ^4^Ludwig Boltzmann Institute for Digital Health and Prevention, Salzburg, Austria; ^5^University Institute of Sports Medicine, Prevention and Rehabilitation, Paracelsus Medical University Salzburg, Salzburg, Austria; ^6^REHA Zentrum Salzburg, Salzburg, Austria; ^7^Institute for Ethics and Law in Medicine, University of Vienna, Vienna, Austria; ^8^Department of Anaesthesia, Intensive Care Medicine and Pain Medicine, Medical University of Vienna, Vienna, Austria; ^9^Institute of Genetics and Animal Biotechnology of the Polish Academy of Sciences, Jastrzebiec, Warsaw, Poland

**Keywords:** continuous non-invasive monitoring, blood pressure, intensive care, validation

## Abstract

**Objectives:**

Continuous non-invasive monitoring of blood pressure is one of the main factors in ensuring the safety of the patient’s condition in anesthesiology, intensive care, surgery, and other areas of medicine. The purpose of this work was to analyze the current patent situation and identify directions and trends in the application of non-invasive medical sensors for continuous blood pressure monitoring, with a focus on clinical experience in critical care and validation thereof.

**Materials and methods:**

The research results reflect data collected up to September 30, 2022. Patent databases, Google Scholar, the Lens database, Pubmed, Scopus databases were used to search for patent and clinical information.

**Results:**

An analysis of the patent landscape indicates a significant increase in interest in the development of non-invasive devices for continuous blood pressure monitoring and their implementation in medical practice, especially in the last 10 years. The key players in the intellectual property market are the following companies: Cnsystems Medizintechnik; Sotera Wireless INC; Tensys Medical INC; Healthstats Int Pte LTD; Edwards Lifesciences Corp, among others. Systematization of data from validation and clinical studies in critical care practice on patients with various pathological conditions and ages, including children and newborns, revealed that a number of non-invasive medical sensor technologies are quite accurate and comparable to the “gold standard” continuous invasive blood pressure monitoring. They are approved by the FDA for medical applications and certified according to ISO 81060-2, ISO 81060-3, and ISO/TS 81060-5. Unregistered and uncertified medical sensors require further clinical trials.

**Conclusion:**

Non-invasive medical sensors for continuous blood pressure monitoring do not replace, but complement, existing methods of regular blood pressure measurement, and it is expected to see more of these technologies broadly implemented in the practice in the near future.

## Introduction

At present, one of the most important tasks facing medicine is to improve the quality of the monitoring process of a patient’s condition and provision timely medical assistance. The speed and quality of the mentioned monitoring process acquire the greatest value in the field of critical care medicine, as a healthcare sector dealing with the extreme degree of manifestation of a pathological process rapidly developing in time.

Monitoring of physiological and biochemical parameters is an important part of the complexity of intensive care medicine and, in a timely manner, indicates a deterioration in the patient’s condition and also helps to assess the effectiveness of the treatment.

As is known in medical practice, continuous monitoring and display of blood pressure measurements is required not only in life-threatening situations, surgical interventions, and anesthesiologic patient support (1).

Hypertension continues to be considered a very powerful predictor of population disability, and its complications, primarily brain stroke and myocardial infarction, largely determine the structure of total mortality. The main task of treating patients with arterial hypertension, and one of the goals during emergency operations in such patients, is to reduce the risk of cardiovascular complications by achieving the target level of blood pressure and organ protection. Monitoring blood pressure can reduce the risk of complications and improve the prognosis of the patients’ outcomes (2, 3).

In this regard, continuous blood pressure monitoring, as opposed to discrete measurement, allows for the early detection of hypo- but also hypertension, the assessment of its duration, the possible reduction of ischemic injuries, particularly to the kidney and myocardium, and the improvement of patient management by lessening the severity of postoperative multiple organ failure and mortality.

Continuous measurement and display of blood pressure is essential in clinical practice to prevent serious disorders and their potentially detrimental consequences. Obviously, non-invasive continuous research methods are of particular importance to critically ill patients.

Following extracardiac surgery, changes in systolic, mean, and pulse pressure in the radial arteries were associated with myocardial and acute renal injury, according to a retrospective analysis by Ahuja et al. (4). Systolic, mean, and pulse pressure hypotension were equally strongly associated with myocardial and renal involvement. Wijnberge et al. have established that intraoperative hypotension during non-cardiac surgery is linked to postoperative cardiac and renal morbidity and mortality (5). Guarracino et al. emphasize that hypotension is common in surgical patients and is associated with hypoperfusion and organ failure (6).

Qualitative monitoring of blood pressure in life-threatening situations, surgical interventions, and anesthesiological support of patients reduces mortality rates and complications, as well as the length of stay in the intensive care unit. It is important to choose a safe and accurate blood pressure monitor to assess blood pressure, since such a device will provide accurate and reproducible measurements.

There are two ways to continuously monitor blood pressure: invasive or non-invasive. Continuous invasive blood pressure monitoring is the “gold standard” in modern anesthesiology and intensive care medicine and has high accuracy. It is per-formed by installing an intra-arterial catheter and connecting it to a monitoring system. It should be noted that continuous invasive blood pressure monitoring has a risk of complications such as trauma, bleeding, infections, thrombosis, embolism, distal ischemia, and pseudoaneurysm formation, to name the most common and severe (7, 8).

The majority of surgical operations employ an oscillometric tonometer to measure blood pressure because it is a simple, non-invasive process. However, this method can only offer intermittent readings, does therefore not really monitor continuously, and does not always accurately and promptly reflect changes in blood pressure. During intraoperative anesthetic treatment, intermittent blood pressure monitoring can miss up to 20% of hypotensive events (9).

Currently, improving the quality of the monitoring process during cardiopulmonary resuscitation is associated with the implementation of modern research methods based on non-invasive medical sensors. One of their main tasks is maximum automation of measurements—thus minimizing the role of a person during the measurement processes (10).

Numerous works by scientists, including Quan X., Tamura T., Meidert A.S., and others, are devoted to issues of achievements in the field of continuous non-invasive blood pressure monitoring (11–13). The authors consider the technical characteristics, accuracy of the devices, their weaknesses and strengths, whereby Meidert et al., postulated that the use continuous invasive blood pressure monitoring with an arterial catheter in patients in critical condition is preferred.

Despite ongoing comparative studies of the accuracy of non-invasive continuous monitoring of blood pressure with other measurement methods, this issue continues to be debated in intensive care in critically ill patients. Methods for continuous non-invasive monitoring of blood pressure have both advantages and limitations, which determines the need for further scientific research in this area.

The development and implementation of an increasing number of medical devices, including those for non-invasive continuous blood pressure monitoring, capable of generating, collecting, analyzing, and transmitting data creates the Internet of Medical Things (IoMT). The IoMT generates information to help improve the speed and accuracy of diagnostics and target treatments more effectively. Additionally, it has the ability to reduce costs, increase effectiveness, and improve patient outcomes. Between 2022 and 2028, the global IoMT market is anticipated to expand at a CAGR of 24.6%.^1^

Patent information is indispensable in the course of scientific research since it allows to assess the novelty and technical level of inventions, thereby reducing research time and costs. Patent analysis may also highlight potential avenues for future research and new perspectives in medical practice (14–16). In world practice, patent analysis is increasingly used for technological forecasting.

Paying tribute to the contribution of scientists to the theory and practice of non-invasive medical sensors for continuous non-invasive monitoring of the condition of patients, it should be noted that the issues of their patent protection and their validation in intensive care require further comprehensive research.

With the present work, we aim to analyze the current patent situation and identify directions and trends in the application of non-invasive medical sensors for continuous blood pressure monitoring, with a focus on clinical experience in critical care and validation thereof.

The review will be of interest to the heads of the departments of patent and invention work, heads of research departments, medical researchers, and clinicians who are willing to deploy IoMT solutions.

## Materials and methods

This study relied on methods such as system analysis, content analysis, scientific synthesis, logical generalization, graphical depiction, and patent research, among others.

The research results are presented as of September 30, 2022. Patent databases, Google Scholar, the Lens database were used to search for patent information. For forming a portfolio of patent documents reflecting the most complete set of solutions in the field of non-invasive medical sensors for continuous monitoring of blood pressure, a search was conducted on the following keyword: non-invasive continuous blood pressure. We used all the keywords with the operator AND. The search was carried out on the title of the patent, abstract, and claims.

The patent search request was limited to the classes of International Patent Classification and Cooperative Patent Classification (17, 18). International Patent Classification and Cooperative Patent Classification codes used in the search are shown in Supplementary Table S1. Some Cooperative Patent Classification codes (e.g., A61B2560/0223, A61B2562/0247, and A61B2562/04) are used for additional information only. The following search query was formulated: (Q1 AND Q2 AND Q3 AND Q4) AND Q5. Q1, Q2, Q3, and Q4 are a combinations of the keywords, while Q5 uses the International Patent Classification and Cooperative Patent Classification codes that match the non-invasive medical sensors for continuous monitoring of blood pressure. Patent studies were conducted according to the guidelines of the World Intellectual Property Organization (19).

A patent search turned up 1,104 patents and 564 simple families containing the keyword “non-invasive continuous blood pressure.” Then we analyzed the resulting array of patents using Patent Classification codes.

As a result, the study of medical sensors for continuous non-invasive blood pressure monitoring covered 573 patents, including 284 families with priority after January 1, 1980. The legal status of the 573 retrieved patents was automatically calculated in the Lens database and is subject to change. At the time of the study, 379 valid patents were identified for the specified search query. The trends of patenting inventions in various technological segments were studied, and the top 20 patent applicants, leading patent security countries, and key inventions were identified.

In order to assess the validation of patented medical sensors for continuous non-invasive blood pressure monitoring, an analysis was made of their registration in the FDA medical device database,^2^ as well as their certification according to ISO standards.

Clinical data of patented medical sensors were studied in the PubMed, Google Scholar, and Scopus databases using their trademarks and commercial names of manufacturers as keywords.

### Current trends in patenting of non-invasive medical sensors for continuous monitoring of blood pressure

**Figure 1:**
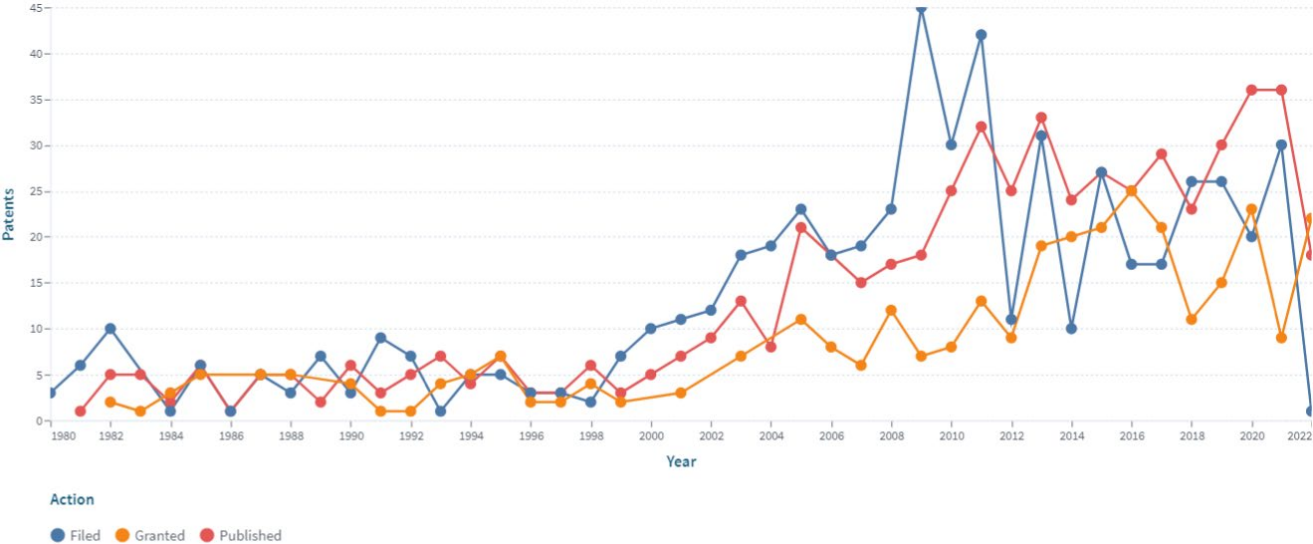
The dynamics of patent and application publications of medical sensors for non-invasive continuously blood pressure monitoring (https://www.lens.org, accessed on September 30, 2022). Since the analysis was performed in September, the presented data for 2022 do not capture the whole respective year.

The analysis of the patent landscape in patent databases (Lens, European Patent Office, US Patent Office) in the field of modern medical sensors for non-invasive continuously measuring blood pressure demonstrates a high interest in the use of non-invasive continuous monitoring. This confirms a significant number of patents for this technology in the databases under study. The dynamics of patent and application publications of medical sensors for non-invasive continuously monitoring blood pressure is shown in Figure 1.

The presented data indicates the presence of an increase in patent activity in this area, which is associated with the prospects for the commercial production of medical sensors for non-invasive continuous monitoring of blood pressure and practical interest in medical practice. It should be noted that the number of patents in this area began to grow in 1999 and has continued to remain at a high level until present, which indicates an unquenchable interest and ongoing research in this area.

Structuring patents by patenting country makes it possible to single out the United States, occupying a leading position, which accounts for 45% of all publications.

The next positions are taken from patents under the Patent Cooperation Treaty, the European Patent Office, and China (21%, 12%, and 12%, respectively). The remaining analyzed countries do not exceed 10% of patent publications.

**Figure 2:**
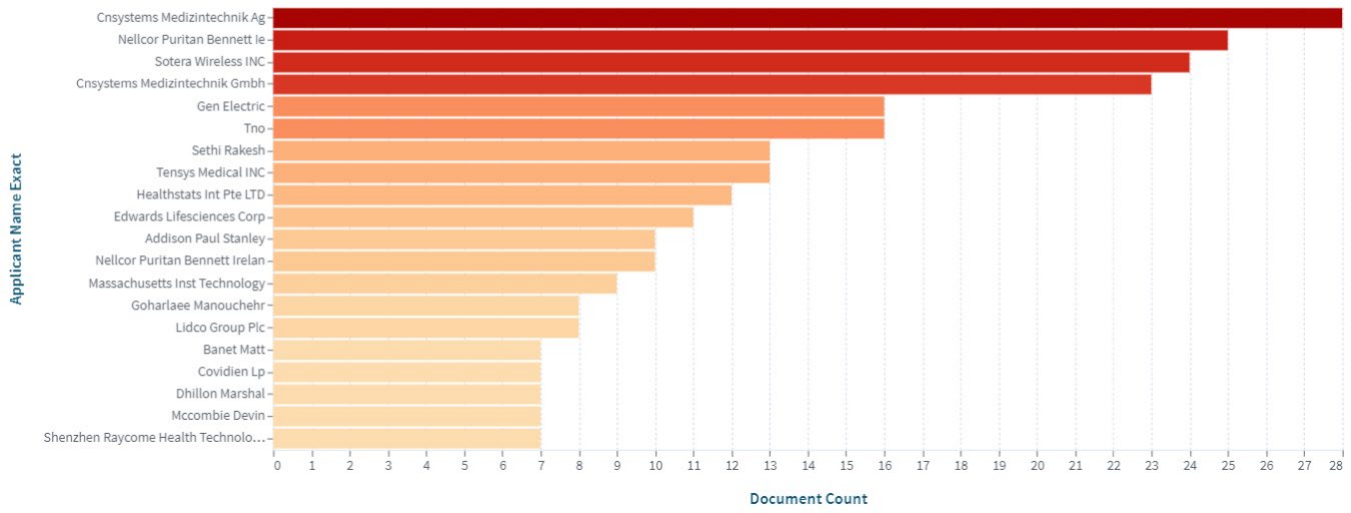
Top applicants of patents in the field of medical sensors for non-invasive continuous blood pressure monitoring (https://www.lens.org, accessed on September 30, 2022).

Among the leading companies, the top 20 applicants of patents are included in Figure 2.

As evident from the data presented in Figure 2, the key players in the intellectual property market are the following companies: Cnsystems Medizintechnik; Nellcor Puritan Bennett Ie; Sotera Wireless INC; Gen Electric; Tno; Sethi Rakesh; Tensys Medical INC; Healthstats Int Pte LTD; Edwards Lifesciences Corp, among others.

According to the WHO’s technical specifications, devices for measuring arterial pressure are divided into invasive and non-invasive group. Non-invasive devices, in turn, are divided into devices with the application of external pressure (cuff-based devices) and without the application of external pressure (cuffless devices). Further, the blood pressure monitoring classification reflects specific quantification methods that are used: pulse transit time, ultrasound or magnetic, photoplethysmography, etc. (1).

Analysis of the patent landscape of non-invasive medical sensors for continuous blood pressure monitoring confirms 2 main directions for non-invasive blood pressure monitoring: cuffed and cuffless devices.

### Cuff-based devices

Cuff-based devices widely use the volume-clamp method first introduced in 1973 by Penaz (20). A little cuff on the patient’s finger has an infrared receiver on one side and a light source on the other. The amount of blood in the finger is determined based on the amount of light absorption. The plethysmograph’s waveform resembles that of the sensor used for invasive blood pressure monitoring. The following are patents in which the volume-clamp technology for cuff-based devices is protected.

#### The CNAP (continuous non-invasive arterial pressure), CNSystems, Austria

The CNAP (Continuous Non-invasive Arterial Pressure; CNSystems, Austria) system is based on the volume-clamp method. The CNAP system uses plethysmography to monitor blood flow in the cuff area of the finger and transmits its fluctuations, transforming them into a constant pulse wave of blood pressure.

**Figure 3:**
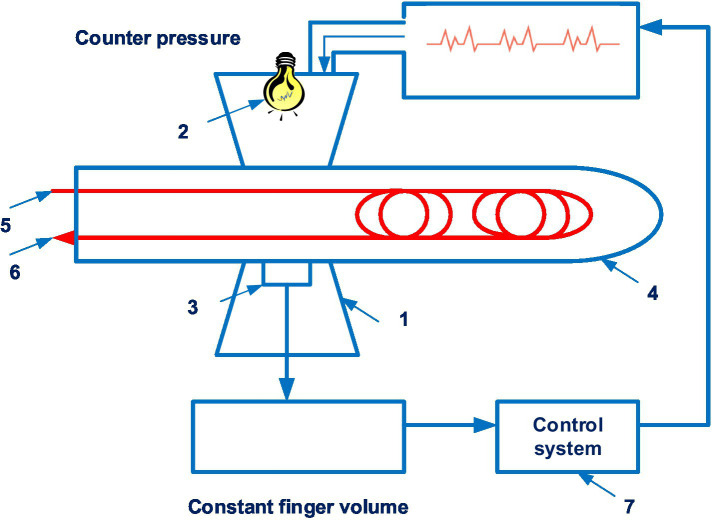
The CNAP system. (1 – finger cuff; 2 – light sources; 3 – light detectors; 4 – a human finger; 5 – an artery; 6 – the vein; 7 – a control system). Diagram modified from reference (21).

The CNAP system is disclosed in patent US 8814800 (21). The patent family includes 18 documents, including patents from Europe, China, Japan, United States, etc. Figure 3 shows the CNAP system (21).

Technically, the device consists of two cuffs mounted on the patient’s fingers and a pressure sensor mounted on the forearm.

Constant pressure in the finger can be accompanied by venous congestion distal to the cuff, the occurrence of “blue finger syndrome,” which can limit the duration of monitoring. To avoid this, two adjacent fingers are sometimes used, which allows the measurement to be switched periodically from one finger to the other, avoiding prolonged venous stagnation. However, this method has limitations in patients with vascular diseases and finger pathology.

Moreover, the arm blood pressure measurement is time-separated from the finger blood pressure measurement and does not address the inherent weaknesses of the arm measurement method used, which therefore results in incorrect blood pressure signal conversion in patients with severe arrhythmia and severe pressure variation (22).

Despite the outlined limitations, as the literature data shows, the CNAP device had reasonable agreement with intermittent oscillometric measurements in the emergency department in 130 patients. The difference between methods for measuring systolic and diastolic blood pressure was −5 mmHg (±22 mmHg) and −2 mmHg (±15 mmHg), respectively. The authors noted that clinically significant hypotensive events can be immediately recognized with continuous arterial pressure monitoring, whereas they are missed or only detected later with intermittent arterial pressure assessment (23). This clearly demonstrates the benefit of continuous blood pressure monitoring.

In cardiothoracic surgery patients (*n* = 51), the CNAP technology showed good agreement when compared with the pulmonary artery catheter (24). Hemodynamic non-invasive measurement using CNAP technology (sensitivity of 76%) seems to be interchangeable with invasive arterial measurements (sensitivity of 81%) in patients (*n* = 47) undergoing major open abdominal surgery (25).

Research by Flick et al. has shown in patients (*n* = 60) undergoing neurosurgical intervention, the absolute and prognostic correspondence between pulse pressure, the invasive blood catheter measured, and the CNAP system was moderate. The predictive agreement between the methods was 82%. An important report is the information provided by Nicklas et al., who established that continuous, non-invasive CNAP monitoring is more effective in determining hypotensive phases during complex gastrointestinal endoscopy of the gastrointestinal tract (*n* = 90) than intermittent blood pressure monitoring (26).

No less relevant is experimental data on the use of CNAP monitoring in children (*n* = 20), whereby it was revealed that some differences in the accuracy of CNAP monitoring were noted during surgical interventions in pediatric practice (27). The correlation coefficient between the arterial cannula and the CNAP device was 0.48, 0.45, and 0.51 for the systolic, diastolic, and mean arterial blood pressure, respectively.

An analysis of data from clinical studies in anesthesiology, intensive care, surgery allows us to conclude that blood pressure measurements using a CNAP device are comparable to invasive measurements in adults. The study on monitoring in pediatrics during surgical interventions revealed some differences in accuracy. More clinical research is needed to determine the feasibility of using this system in children.

#### ClearSight (Nexfin), Edwards Lifesciences, United States

Of note is the ClearSight (Nexfin) device (Edwards Lifesciences, United States), which is also based on the volumetric clamp technique and is presented in patent application WO2020176207 (28). In an international patent application, Edwards Lifesciences claimed several countries for patenting. A finger cuff is disclosed that includes an optical source and a sensor pair for measuring a plethysmography signal from a finger artery. Supplementary Figure S1 shows a flow diagram for non-invasive blood pressure monitoring by the ClearSight (Nexfin) (28).

The effectiveness of the ClearSight system has been demonstrated in patients undergoing surgery. In 54 patients, invasive intra-arterial blood pressure monitoring during heart surgery was compared to non-invasive blood pressure measurement utilizing the ClearSight vascular unloading technology (Edwards Lifesciences Corp) (29). The percentage error was calculated as 1.96 SDbias/mean reference method (invasive arterial blood pressure measurement). The mean arterial, systolic, and diastolic pressures measured by ClearSight have corresponding percentage errors of 25.95, 26.77, and 34.16%. Thereby, if a percentage error is less than 30%, the ClearSight method is considered equivalent to the reference method. The ClearSight system is recommended by the authors as a viable choice for hemodynamic monitoring during anesthetic induction.

Researchers investigated the effect of ClearSight monitoring on blood pressure stability during general anesthesia in 160 orthopedic patients in a randomized trial (30). The authors conclude that continuous monitoring contributes to the stabilization of blood pressure in the study population.

A comparative evaluation of methods for assessing cardiac output using the ClearSight system and the pulmonary artery catheter bolus thermodilution method was carried out in a multi-center clinical study (*n* = 125) in China (31). It has been established that these research methods in cardiac surgery patients are comparable.

Also, like the CNAP system, the ClearSight system was more effective for the identification of hypotensive reactions during gastrointestinal endoscopy (*n* = 20) (32). Moreover, the ClearSight system has been used to provide 56 patients who underwent gynecologic oncologic surgery with real-time and precise predictions of impending arterial hypotension (33).

Comparative studies of the ClearSight system (*n* = 40) and traditional oscillometric blood pressure monitoring to reduce the incidence of hypotension during caesarean sections was investigated by Juri et al. (34). The ClearSight system had acceptable accuracy, and its application resulted in less maternal hypotension and nausea.

It was claimed that, when performing a caesarean section (*n* = 31) under a central neuraxial anesthetic while using ClearSight, a non-invasive continuous blood pressure monitor, the difference between the diastolic and mean arterial blood pressure and the reference method is acceptable (35). Patients with severe aortic stenosis having elective transcatheter aortic valve replacement were monitored for mean blood pressure, systolic and diastolic blood pressure with the ClearSight device (*n* = 20). The device displayed high levels of accuracy, consistency, and precision (36). The ClearSight System was evaluated in 18 patients during cardiovascular surgery (37). The authors recommend using this system as an alternative to mean radial arterial pressure.

However, the correlation of measurements using the ClearSight system and an arterial catheter was moderate in obese patients after laparoscopic bariatric surgery (*n* = 44). It has been established that the ClearSight system-derived cardiac index is insufficiently accurate in patients undergoing off-pump coronary artery bypass graft surgery who have a lower ejection fraction (38, 39). However, in patients with low ejection fraction, ClearSight’s system accuracy and trending capabilities for mean arterial pressure were clinically acceptable.

Bartels et al. conducted an analysis of studies of CNAP and ClearSight (Nexfin) systems with direct arterial catheterization (8). The studies were carried out in the period 2009–2015 and comparisons were done using various statistical methods. The authors conclude that, overall, the obtained data suggest performance comparable to oscillometric methods, although there are no large retrospective trials to confirm prospective observations in real clinical settings.

A widely accepted standard of evidence-based medicine is a meta-analysis of the results of multiple studies. The meta-analysis of 10 studies showed that photoplethysmography technologies (CNAP device, CNSystems, Austria; Clearsight, Edwards Lifesciences, US) are poorly interchangeable with bolus thermodilution in ICU and cardiac surgical settings (40). It should be noted that, according to World Health Organization’s (WHO) technical specifications, an effective regulatory system is needed to ensure independent validation of the accuracy of novel blood pressure measuring devices (1).

It should be noted that the presented meta-analysis was carried out for the period 2010–2018. A number of randomized and controlled clinical trials in recent years have shown that Clearsight allows preventive clinical decisions in cardiac surgery, orthopedics, GI endoscopy, and caesarean section. The accuracy of the obtained BP values using the ClearSight system was moderately consistent with the data of invariant measurement in obese patients.

#### Lidco hemodynamic monitor, Lidco group Plc, England

**Figure 4:**
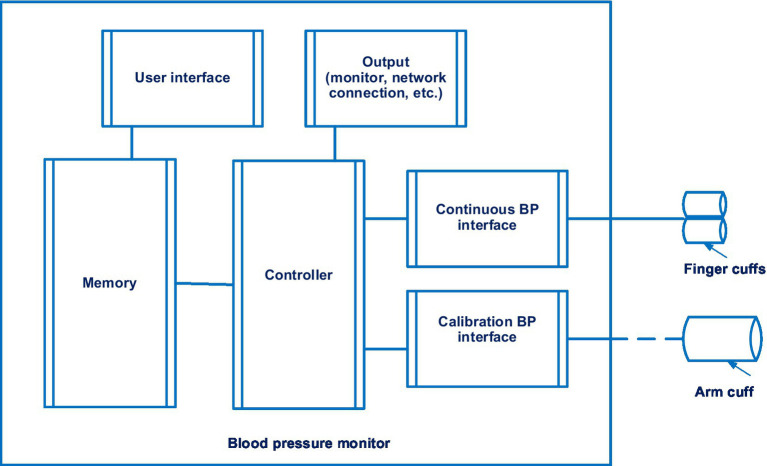
The system for continuously monitoring blood pressure in accordance with the Lidco invention. Diagram modified from reference (41).

The Lidco hemodynamic monitor for non-invasive continuous monitoring of blood pressure also merited consideration. It is disclosed in patent EP 2914168 A1 (41). 14 documents are in this patent family, including patents from Europe, China, Japan, United States, etc. The Lidco hemodynamic monitor also uses the volume-clamp method by Penaz. Figure 4 shows a system for continuously monitoring blood pressure in accordance with the Lidco invention.

A feature of Lidco’s invention is the presence of a controller that measures blood pressure twice. If the first blood pressure measurement exceeds a predetermined threshold, a second blood pressure measurement will be initiated.

In a prospective study, less than half of the postoperative patients (39%) kept the Lidco device in place until the end of a 12 h period of continuous postoperative follow-up (*n* = 104). According to the authors, the technology must be improved for this indication. However, with the Lidco device, numerous instances of early postoperative hypotension have been found (42).

According to the presented data from a clinical study, the use of Lidco contributes to clinical decision support. However, there is a limitation in the movement of patients when using it.

#### Finapres/Finometer, Finapres medical systems, Netherlands

Another cuff device, the work of which is based on the classical volumetric clamp method, is the Finapres (Finapres Medical Systems, Netherlands). Finapres/Finometer technology is disclosed in patent application EP 2319408 (43). 4 documents are in this patent family, including patents from Europe, Japan, United States, etc.

**Figure 5:**
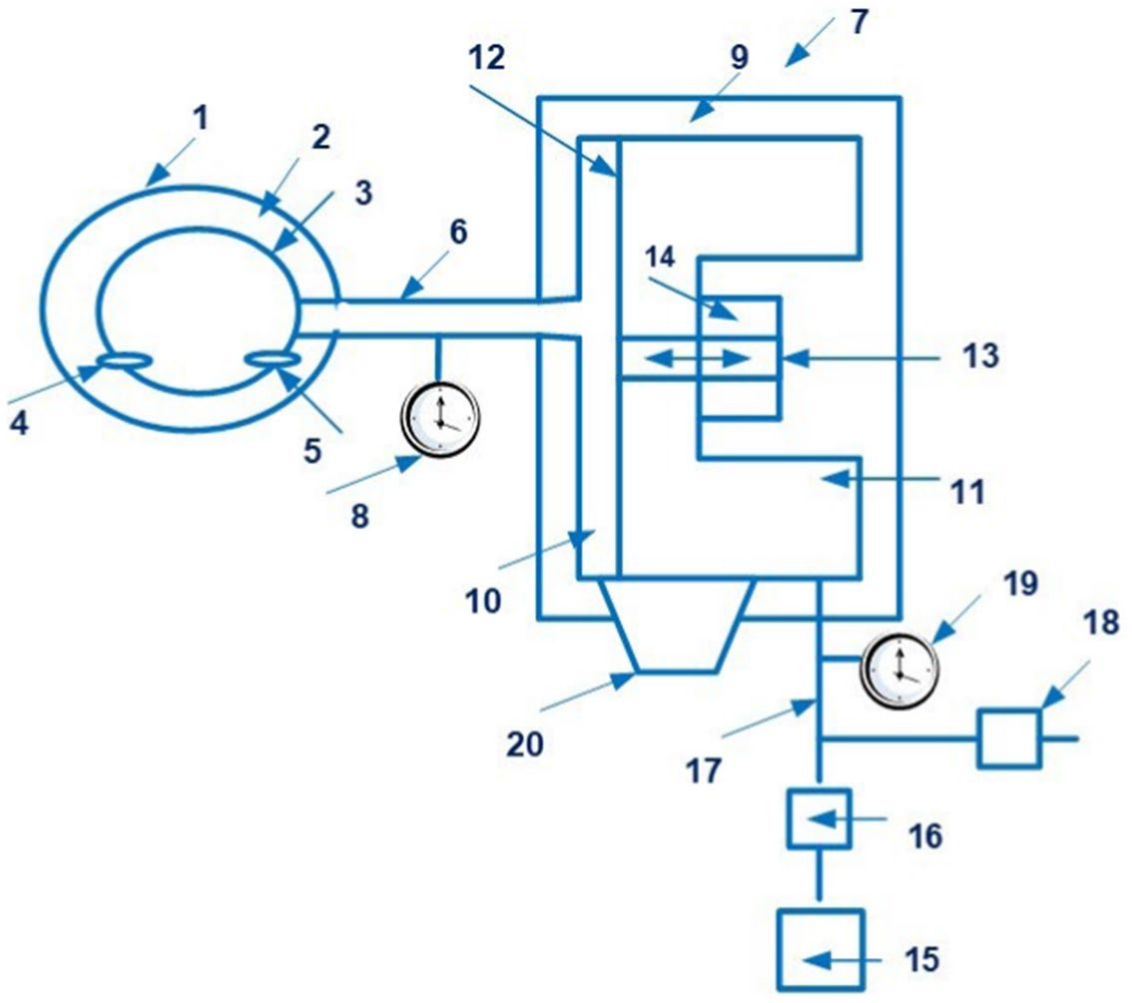
A schematic view of a Finapres device for controlling the pressure in the finger cuff (1 – finger cuff; 2 – outer sleeve; 3 – the inflatable bladder; 4 – an infrared LED; 5 – an infra-red sensor; 6 – a line; 7 – a device for controlling the pressure in the bladder; 8 – a pressure sensor; 9 – the housing; 10, 11 – chambers; 12 – the membrane; 13 – a rod; 14 – a coil; 15 – an air pump; 16 – a valve; 17 – a line; 18 – an exhaust valve; 19 – a pressure sensor; 20 – a bypass channel). Diagram modified from reference (43).

This device also uses photoplethysmography based on the determination of relative changes in blood volume in blood vessels in the dermis and subcutaneous fat. Figure 5 shows a schematic view of a Finapres device for controlling the pressure in the finger cuff.

There is data from studies of the Finapres system under conditions of emergency care. Cardiovascular monitoring was performed using Finapres during early mobilization after breast cancer surgery (*n* = 24) (44). Orthostatic intolerance 30 min after surgery was rare (4%).

Data from clinical randomized trials of Finapres in intensive care and anesthesiology have not been identified over the past 5 years.

#### The VitalStream, Caretaker Medical, United States

**Figure 6:**
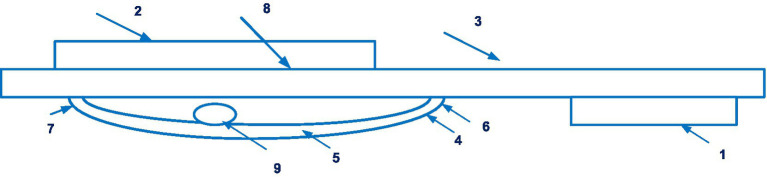
The cuff of the VitalStream (1 – the hook section, 2 – the loop section, 3 – the substrate member, 4 – an inflatable membrane, 5 – the periphery of the membrane, 6, 7 – edges, 8 – interior region, 9 – tube). Diagram modified from reference (45).

Systems and methods for indirect, oscillometric, digital blood pressure monitoring that provide self-calibration to obtain absolute blood pressure values are used in the VitalStream device. It is described in patent application WO 2019/010416 A1 (45). 8 documents are in this patent family, including patents from Europe, Japan, United States, etc. Figure 6 shows the cuff of the VitalStream.

The VitalStream sends vital sign data from a wearable device to a mobile app, cloud portal, or integrates with other monitoring systems.

Experimental data is available on the accuracy of the CareTaker device in patients. There are data from comparative studies in patients undergoing major intra-abdominal surgery (*n* = 24), continuously non-invasive blood pressure measurement with the CareTaker, and invasive intra-arterial pressure measurement (46). The data obtained had a high correlation. The authors noted the need for further large-scale studies.

In intensive care unit patients, the accuracy of non-invasive blood pressure measurement with the CareTaker device and invasive arterial blood pressure measurement was investigated (*n* = 34). The overall correlation between complexes was 0.99 (47). The authors conclude that non-invasive blood pressure monitoring using the CareTaker device is possible. A comparability between the results from both methods is attained in abdominal surgery over a significant fraction of time overall (48).

Thus, clinical data support the possibility of using CareTaker to monitor hypotensive conditions in the postoperative period.

### Cuffless devices

The patenting of cuff-free devices is connected to the second area of advancement in the use of non-invasive medical sensors for continuous monitoring. Solutions for continuous monitoring without cuffs are based on methods for calculating an impulse’s transit duration, examining a pulse wave, etc. One of the principles that is used in cuffless devices is the applanation tonometry of the radial artery. Applanation tonometry is based on the registration of a pulse wave during the “flattening” of the artery. A highly sensitive sensor presses on the radial artery of the hand in the area of the wrist and records the values of the pulse and blood pressure.

#### The T-line system, Tensys Medical, United States

A device enabling automatic applanation tonometry of the radial artery is the T-Line TL-200 system (Tensys Medical, United States) disclosed in patents EP 1538971, US 10952675 (49, 50). 30 and 13 documents are in these patent families, respectively. These patents are covered in Canada, Europe, Japan, Korea, United States, etc.

Supplementary Figure S2 is a logical flow diagram illustrating the process of maintaining optimal applanation level.

There is validation data for the T-Line system in intensive care. In 28 patients receiving intensive care, a femoral arterial catheter and the T-Line TL-200 system were used to measure blood pressure simultaneously (51). A low error was found for mean arterial pressure for the T-Line TL-200 system compared to mean arterial pressure assessed using a femoral arterial catheter. The authors conclude that blood pressure measurement with the T-Line TL-200 is generally feasible for patients in the intensive care unit. The device’s high limits of agreement preclude its use as the sole source of blood pressure information in critically ill patients who are unstable.

It has been presented data on the comparative measurements of cardiac output using the T-Line system and intermittent thermodilution of the pulmonary artery using a pulmonary artery catheter in patients (*n* = 50) after cardiothoracic surgery (52). The percentage error was 34%. The level of compliance was 95%.

It has also been established that in 24 patients in the intensive care unit, mean arterial pressure and diastolic blood pressure measured by the T-Line system demonstrated clinically acceptable agreement with invasive determination using a radial arterial catheter. The accuracy of systolic blood pressure measurement needs further improvement (53). Similar results using the T-Line system were obtained in patients (*n* = 23) with multiple organ dysfunction syndrome (54).

It has been also reported that the T-Line system displayed to be unreliable in intensive care patients (*n* = 31) with severe comorbid heart disease (55). The application of T-Line system is noted to be limited in morbidly obese patients undergoing bariatric surgery after extensive operations on the gastrointestinal tract. Further studies are needed to confirm the possibility of using this system in patients with morbid obesity (56).

Thus, the T-Line system using applanation tonometry is applicable to patients after cardiac surgery. It should be noted that the T-Line system’s use is restricted in patients with multiple organ dysfunction syndrome or morbid obesity. It should be noted that Tensys Medical is committed to improving patient safety by developing a new device for continuous non-invasive hemodynamic monitoring. The TL-300 measures blood pressure continuously and has the ability to view trend data for up to 12 h.

#### Boppli, PyrAmes, United States

**Figure 7:**
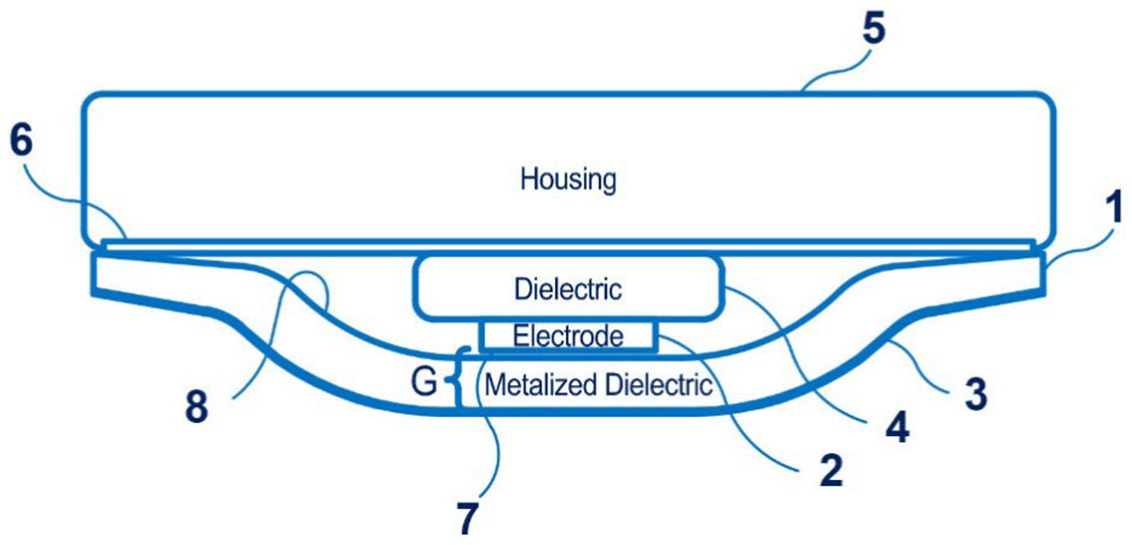
The proximity sensor of the Boppli device (1 – a first dielectric layer; 2 – a sensing electrode; 3 – an electrically conductive layer; 4 – the second dielectric layer; 5 – the housing; 6 – an adhesive; 7 – the surface of the sensing electrode; 8 – the first dielectric layer; a distance G separates the surface of the sensing electrode 2 from the electrically conductive layer). Diagram modified from reference (57).

Of particular note are the sensors for the Boppli device (PyrAmes, US), which are disclosed in patent application WO 2021/067893 A1 (57). Four documents are in this patent family, including patent documents from Europe, China, and Korea. The device is designed for continuous non-invasive blood pressure measurement, including in newborns. It can be applied to the arm or leg of the patient, which is convenient for newborns. This capacitive sensor technology can collect pulse shape data. Figure 7 shows the proximity sensor of the Boppli device.

Non-invasive blood pressure monitoring technologies cannot completely replace permanent, invasive blood pressure monitoring in children during surgical interventions. Ilies et al. discuss that the reason for this lies in the low efficiency of these devices during periods of sharp fluctuations in blood pressure and hypotension (58). In their study, van Wijk et al. noted that modern medical sensors can be used as additional tools along with standard monitoring in children to improve perioperative safety (59).

The first studies of the Boppli device (PyrAmes, US) in 120 patients aged 4 to 87 years and 16 patients aged 1 to 8 days from intensive care units, pediatric intensive care units, and cardiovascular intensive care units at Stanford University Medical Center show the good accuracy of the device (11). The pulse waveform obtained using capacitive sensor technology showed a strong correlation with blood pressure values obtained through an arterial catheter.

The FDA has awarded the Boppli device a Breakthrough Device Designation (BDD), which addresses an unmet need for blood pressure monitoring in critically ill children when an invasive arterial catheter cannot be used due to the risk of complications and adverse effects. The FDA BDD program is designed to promote the development and accelerate the review of advanced medical device technologies. The data presented show that the Boppli application has positive results in various age groups.

#### Aktiia bracelet, Aktiia SA, Switzerland

Another promising technology in the development of medical sensors for non-invasive continuous blood pressure monitoring, involving the acquisition and analysis of a pulse wave, is a cuffless device developed by Aktiia (Switzerland), disclosed in patent application WO 2021/229276, US2021315464 (60, 61). Aktiia claimed several countries for patenting, including Europe, China.

**Figure 8:**
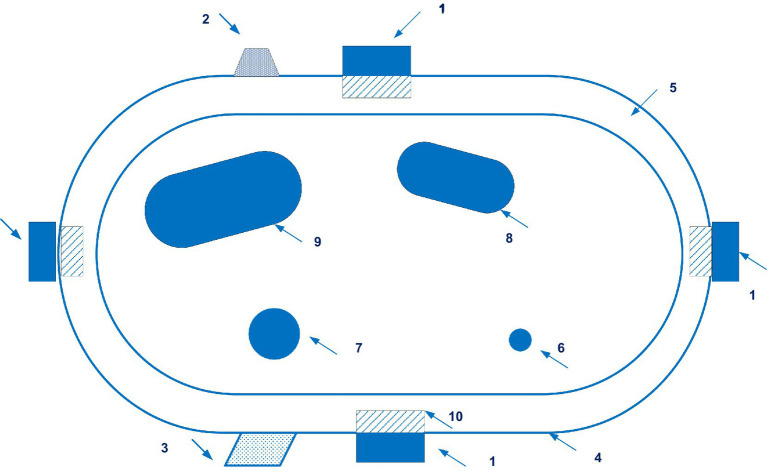
A configuration of a wearable device of the Aktiia. (1 – pulsatility sensing unit; 2 – motion sensor; 3 – geolocation means; 4 – armband; 5 – wrist’s skin; 6 – ulnar artery; 7 – radius artery; 8 – ulna bone; 9 – radius bone; 10 – capillary bed). Diagram modified from reference (61).

Figure 8 illustrates a configuration of a wearable device of the Aktiia.

The validation of the optical bracelet Aktiia in various positions of the body for continuous monitoring of blood pressure in 91 patients with blood pressure levels from low to hypertensive was carried out (62). It is shown that the bracelet Aktiia is able to generate blood pressure readings with a high probability in motionless conditions in both sitting and lying positions. An effective measurement is not provided when the arm is moved and a few seconds after the cessation of any physical activity.

Vybornova et al. reported that the accuracy and precision of the Aktiia bracelet when used for 1 month (*n* = 86) in an outpatient setting met test criteria 1 and 2 of the ISO81060-2 standard in the sitting position (63).

Comparative studies of systolic and diastolic blood pressure measurements using the Aktiia bracelet were carried out, with invasive measurements performed in patients (*n* = 31) with radial artery catheterization in the intensive care unit (64). The authors conclude that non-invasive monitoring with the Aktiia bracelet can replace more traditional methods for assessing blood pressure.

Therefore, the clinical evidence indicates that the Aktiia bracelet meets the acceptance criteria of the ISO 81060-2 standard, which makes it eligible for clinical use.

#### Biobeat, Biobeat, Israel

Another original invention is used by the developers of the Biobeat monitoring device presented in patent application WO 2021/250681 A1 to solve the problem of continuous non-invasive blood pressure measurement (65). Two documents are in this patent family, including patent documents from United States.

**Figure 9:**
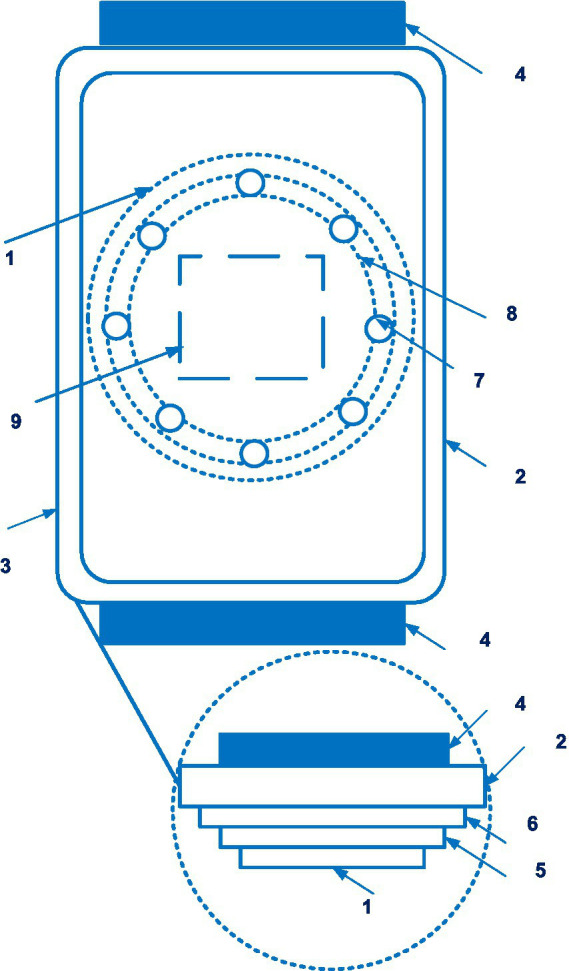
A sensor device of the Biobeat (1 – the sensor device; 2 – a housing; 3 – a display; 4 – adjustable strap; 5 – battery; 6 – substrate; 7 – the light emitters; 8 – reflectors; 9 – the light detector). Diagram modified from reference (65).

Biobeat devices also use the plethysmography method and several light sources of different wavelengths. As a result, users receive reliable results. Figure 9 shows a sensor device of the Biobeat.

A positive effect of using the Biobeat device during labor based on reflective photoplethysmography has been reported (*n* = 81) (66). Further research shows that vital signs obtained with photoplethysmography-based devices can be affected by subcutaneous fat and skin color. Of interest is a comparative study of blood pressure measurement accuracy between the Biobeat device and cuff-based blood pressure measurements in 1057 patients (67). Similar and high coincidences were found between blood pressure measurements by different methods in different groups by sex, body mass index, and skin color, which is of great interest for medical practice.

Kachel et al. compared blood pressure measurements with a Biobeat device and with arterial line sensors in 10 patients immediately after cardiac surgery (68). The authors conclude that Biobeat devices provide a higher level of accuracy in cardiac parameters compared to arterial line sensors in patients undergoing cardiac surgery.

A prospective, comparative clinical study (*n* = 7) was conducted in the general intensive care unit of cardiac output measurements obtained with the Biobeat photoplethysmography-based non-invasive chest patch and a pulse circuit cardiac output device (69). The values obtained by thermodilution and photoplethysmography had a high correlation (*r* = 0.906). The authors conclude that the device has enhanced monitoring capabilities in a variety of clinical settings without the complications associated with invasive devices.

Thus, the Biobeat provides accurate non-invasive monitoring of blood pressure during childbirth and cardiac surgery, uses a cloud platform. The measurement results were not influenced by body mass index or skin color.

#### ViSi Mobile, Sotera Wireless, US

Sotera Wireless has created a non-invasive continuous blood pressure monitoring system disclosed in patent EP 3102097 (70). Eleven documents are in this patent family, including patent documents from China, Europe, and United States.

The invention discloses a system that monitors a variety of indicators, including blood pressure, by estimating the pulse wave velocity. Supplementary Figure S3 shows a flow chart for measuring cNIBP, SpO2, respiration rate, heart rate, temperature, and motion according to the ViSi Mobile.

A comparative evaluation of monitoring using the ViSi Mobile device (Sotera Wireless) and HealthPatch (Vital Connect) was carried out in a pilot study on 20 patients (71). Measurement inaccuracies were detected, which were due to connection errors.

A prospective 5 month pilot study of the ViSi Mobile device was conducted in a neurological/neurosurgical department for adults (72). Against the background of the use ViSi Mobile intensive care unit transfers per 1,000 discharged patients decreased by 25%. Unplanned patient deaths in the unit decreased as well, but the difference is statistically unreliable in this study.

In a prospective, blinded, observational study (*n* = 312), including data from studies NCT02156154 and NCT02996227, the ViSi Mobile system was used to monitor blood pressure in patients after abdominal surgery (73). The authors conclude that continuous blood pressure monitoring detects hemodynamic disturbances more effectively and helps in timely intervention and treatment.

Thus, ViSi Mobile promotes recognition and detects the deterioration of the patient’s condition. It allows doctors to carry out timely and effective interventions.

#### Somnotouch NIBP, Somnomedics GmbH, Germany

**Figure 10:**
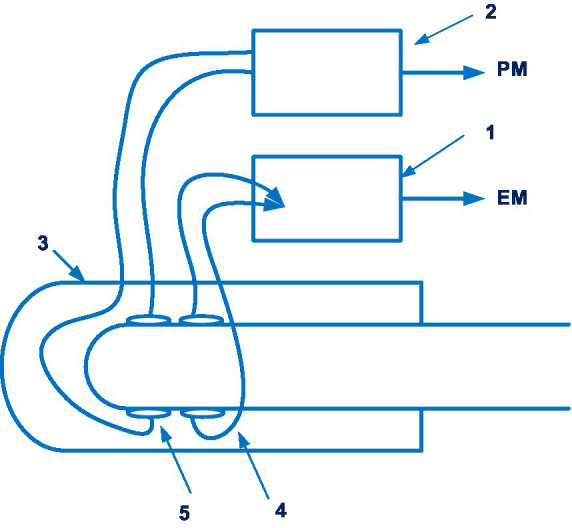
A module of the Somnotouch-NIBP device (1 – ECG sub-unit; 2 – a pulse-oximeter sub-unit; 3 – a housing; 4 – an ECG sensor; 5 – a pulse sensor; a pulse measurement signal (PM); the electrical measurement signal (EM)). Diagram modified from reference (74).

To obtain a blood pressure measurement by the Somnotouch-NIBP device (Somnomedics GmbH, Germany) presented in patent EP 1704820 B1 (74), the pulse transit time is estimated. Three documents are in this patent family, including patent documents from Europe and United States. Figure 10 shows a module of the Somnotouch-NIBP device.

In a prospective study of 71 patients over 24 h, a significant difference was found between the measurements of different devices (75). Blood pressure measurements using the Somnotouch-NIBP device showed higher systolic and diastolic blood pressure. The authors conclude that further research is needed to determine the effectiveness of this device.

Validation of Somnotouch-NIBP in compliance with ESH (European Society of Hypertension) protocols was satisfactorily completed. However, after the initial calibration, this validation only requires 30 min of testing. When certifying cuff-free blood pressure measurement equipment, the Lancet Commission on Hypertension Group suggests using the current ISO recommendation (76–78).

#### BPro, HealthStats, Singapore

**Figure 11:**
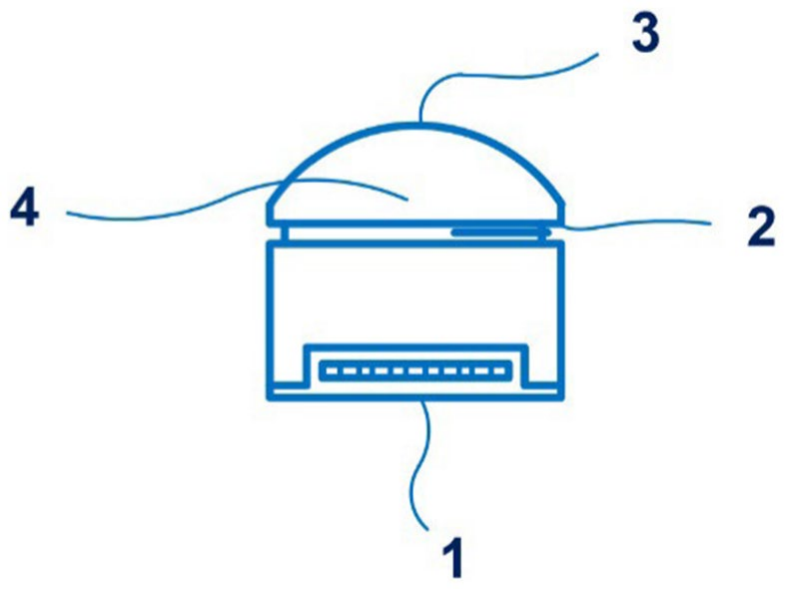
A side view of the sensor of the BPro system (1 – a transducer; 2 – the diaphragm; 3 – a plunger; 4 – a layer of gel). Diagram modified from reference (79).

The BPro system (HealthStats, Singapore) is based on the patented applanation tonometry blood pressure measurement technology (patent HK 1077185 A1) (79). 25 documents are in this patent family, including those from Australia, Europe, Israel, China, New Zealand, Japan, etc. Figure 11 shows the sensor of the BPro system.

A comparison was made between blood pressure measurement with the BPro system and an oscillometric cuff device in patients with diabetes mellitus (*n* = 25). The authors conclude that tonometric and cuff blood pressures are comparable (80).

The BPro device was validated (*n* = 45) for measuring blood pressure during pregnancy and gestational hypertension (81). BPro has been found to be accurate in pregnancy. In women with preeclampsia, BPro also met the validation criteria (−2.7 ± 7.1 and 0.3 ± 4.7 mmHg for SBP and DBP, respectively) of the universal standard protocol, but the differences were greater than in the gestational hypertension and normotension groups.

A study of 28 patients undergoing elective surgery found an inaccurate agreement between BPro medical sensor measurement and invasive blood pressure measurement (82).

No other studies on the use of BPro system in intensive care or anesthesiology have been identified. BPro system is certified in accordance with ISO 81060-2, ISO 81060-3, ISO/TS 81060-5. It should be used with caution in patients with confirmed preeclampsia.

#### SentiCor-100, Sensifree, United States

**Figure 12:**
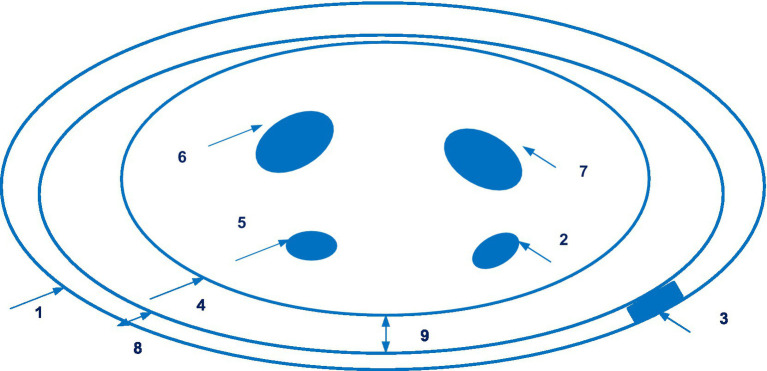
The wearable device (Sensifree) for measuring arterial blood pressure (1 – wristband; 2 – radial artery; 3 – sensor; 4 – the cross-section of a wrist; 5 – the ulnar artery; 6, 7 – bones in the wrist; 8 – the thickness of the wrist band; 9 – the shortest distance between the surface of the wrist and one point along the wristband). Diagram modified from reference (84).

The company Sensifree has launched a device for continuous non-invasive blood pressure monitoring based on photoplethysmography. This technology is disclosed in patent EP 3169227 A1 (83), and application US 2020/0253564 A1 (84). EP 3169227 patent families contain five documents, including patent documents from China, Europe, and United States. Figure 12 shows the wearable device (Sensifree) for measuring arterial blood pressure.

There is data available from a single-centre, non-randomized comparative study of the use of the Sensifree medical sensor in 14 healthy volunteers with invasive arterial line. It was concluded that the Sensifree device signal was significantly more similar to the arterial line signal than the photoplethysmography sensor signal from the pulse oximeter (85).

The proper accuracy of SentiCor-100 is evidenced by the fact that the manufacturer received an EU in compliance with the new EU MDR (August 31, 2022).

#### SeismoWatch, Georgia Tech Research Institute and Northwestern University, United States

**Figure 13:**
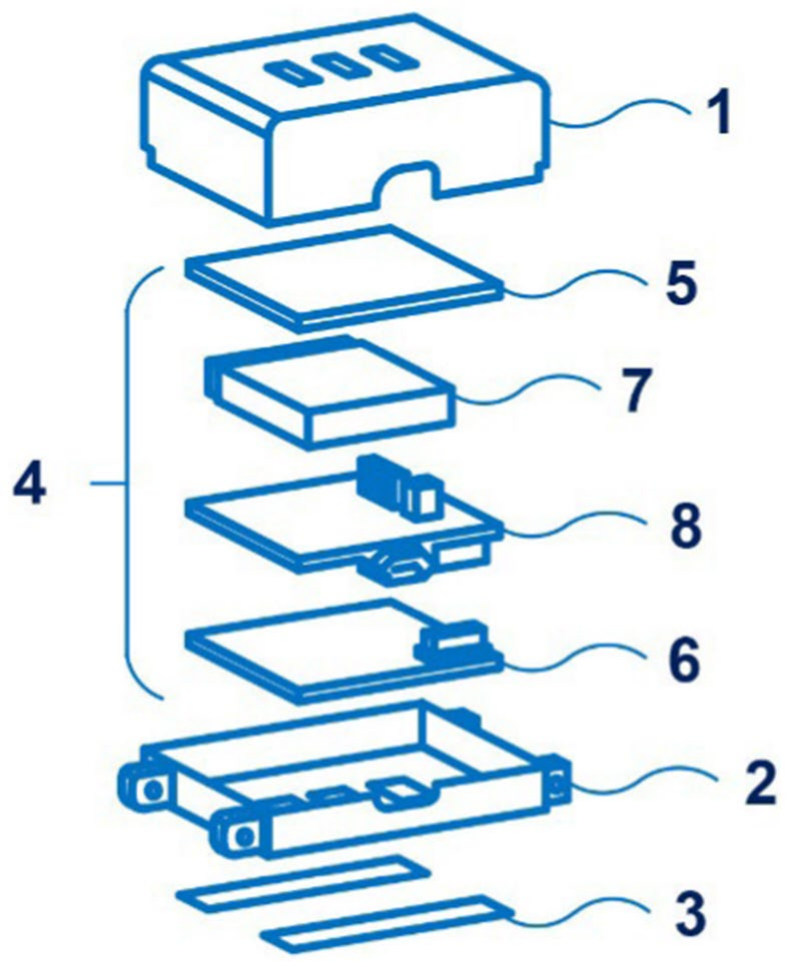
An exploded view of the wearable device SeismoWatch (1 – first surface; 2 – s surface; 3 – sensor; 4 – processor; 5 – first PPG board; 6 – s PPG board; 7 – a battery; 8 – a mainboard). Diagram modified from reference (86).

Of interest is also the development of the SeismoWatch bracelet by inventors from the Georgia Tech Research Institute and Northwestern University. Its principle is disclosed in the application WO 2021/188878 and is based on a combination of photoplethysmography and seismocardiography (86). Applicants claimed several countries for patenting. Figure 13 shows an exploded view of the wearable device SeismoWatch.

Seismocardiography is a method of measuring the vibrations of the chest caused by the heartbeat. The bracelet, applied to the sternum for a short time (about 15 s), registers the fluctuations of the chest associated with heart contractions. This data allows wrist photoplethysmography to calculate the pulse transit time, on the basis of which blood pressure is calculated. It should be noted that the instrument requires periodic calibration.

The pilot study showed that the SeismoWatch bracelet significantly outperformed the conventional pulse arrival time system based on wearable blood pressure assessment (87).

Clinical trials of the SeismoWatch bracelet for non-invasive blood pressure measurement in anesthesiology and intensive care therapy have not been conducted to date, but the available results indicate the prospect of this system. The device is under research and development.

## Discussion

An analysis of data from 1980 to the end of September 2022 of the patent landscape of non-invasive medical sensors for continuous blood pressure monitoring suggests a significant increase in interest in the development and implementation of new devices in medical practice, especially in the last 10 years.

According to the received data we can conclude about positive dynamics of the patenting, including the following companies Cnsystems Medizintechnik; Nellcor Puritan Bennett Ie; Sotera Wireless INC; Gen Electric; Tno; Sethi Rakesh; Tensys Medical INC; Healthstats Int Pte LTD; Edwards Lifesciences Corp, among others. It has been established that patents for cuffed devices have earlier priority dates (e.g., 2000–2019), to a greater degree. Priority dates for inventions related to patenting uncuffed devices are more recent (e.g., 2016–2021), to a greater degree. It should be noted that medical sensors for continuous pressure monitoring are characterized by comprehensive patent protection. Devices are usually protected by several patents. The patents shown above are only for illustration and do not constitute a limitation. There are a number of other inventions in the field of non-invasive medical sensors for continuous monitoring of blood pressure. In the last two decades, the scientists have actively created and patented developments in this direction.

The value of blood pressure as a major predictor of the development of cardiovascular and postoperative complications explains the interest in investigating medical sensors for continuous blood pressure monitoring, especially in the intensive care unit. As a result, it is possible to identify early hypo- and hypertension cases, assess their duration, and improve patient management by lowering the severity of postoperative insufficiency and mortality. Non-invasive medical sensors for continuous blood pressure monitoring also allow for the avoidance of the risks of complications such as trauma, bleeding, infections, thrombosis, embolism, distal ischemia, among others, that may occur after the use of invasive medical sensors.

The overview of the literature showed that for patented non-invasive medical sensors for continuous blood pressure monitoring, a number of studies have been conducted in the intensive care unit, resuscitation, and surgery on patients of various ages, including children and newborns, suffering from various pathological conditions.

The characteristics of cuff-based devices for non-invasive continuous monitoring of blood pressure are listed in Table 1.

**Table 1:** The characteristics of cuff-based devices for non-invasive continuous monitoring of blood pressure.

Device, company name, country	Measurement technology, device location	Disadvantages	Validation^*^	Status of the device
CNAP, CNSystems, Austria	Volumetric clamp technique plethysmography, finger	The occurrence of “blue finger syndrome,” limitations in patients with vascular diseases, and finger pathology	ISO 81060-2, ISO 81060-3, ISO/TS 81060-5	Approved FDA and commercialized
ClearSight (Nexfin), Edwards Lifesciences, United States	Volumetric clamp technique plethysmography, finger	After 8 h of continuous monitoring on a single finger, the finger cuff should be reapplied to another finger.	ISO 81060-2, ISO 81060-3, ISO/TS 81060-5	Approved FDA and commercialized
Lidco Hemodynamic Monitor, Lidco Group Plc, England	Volumetric clamp technique, Finger	Alternating between the two cuffs, limiting hand movement	ISO 81060-2, ISO 81060-3, ISO/TS 81060-5	Approved FDA and commercialized
Finapres/Finometer, Finapres Medical Systems, Netherlands	Volumetric clamp technique plethysmography, finger	Alternating between the two cuffs, limiting hand movement	ISO 81060-2, ISO 81060-3, ISO/TS 81060-5	Approved FDA and commercialized
The VitalStream, Caretaker Medical, United States	Pulse decomposition analysis, finger	Alternating between the two cuffs, limiting hand movement	ISO 81060-2, ISO 81060-3, ISO/TS 81060-5	Approved FDA and commercialized

^*^https://www.accessdata.fda.gov/scripts/cdrh/cfdocs/cfRL/rl.cfm. ^**^ISO 81060-2 Third edition 2018-11 Non-invasive sphygmomanometers – Part 2: Clinical investigation of intermittent automated measurement type [Including: Amendment 1 (2020)]; ^***^ISO 81060-3 First edition 2022-12 Non-invasive sphygmomanometers – Part 3: Clinical investigation of continuous automated measurement type; ^****^ISO/TS 81060-5 First edition 2020-02 Non-invasive sphygmomanometers – Part 5: Requirements for the repeatability and reproducibility of NIBP simulators for testing of automated non-invasive sphygmomanometers.

Analysis of cuff-based devices for non-invasive continuous blood pressure monitoring allows the following briefly characterization. The CNAP device showed promising results in the emergency department, cardiothoracic surgery, and complex gastrointestinal endoscopy of the gastrointestinal tract, whereby cardiac monitoring in pediatric practice using CNAP requires further research.

The ClearSight device demonstrated the ability to quickly detect hemodynamic disorders in cardiac surgery, orthopedics, gastrointestinal endoscopy, and caesarean sections. However, the correlation of measurements using the ClearSight system and an arterial catheter was moderate in obese patients, as well as in those who underwent coronary artery bypass surgery with a lower ejection fraction.

The Lidco monitor has been found to detect numerous cases of early postoperative hypotension. But, at the same time, it was noted that there is a restriction in the movement of patients when using it.

Data from clinical randomized trials of the Finapres device in intensive care and anesthesiology over the past 5 years have not been identified. Further research is needed to validate the latter device in such setting. The device developed by Caretaker also showed a high correlation with invasive blood pressure measurements in the intensive care unit.

An important aspect of using non-invasive blood pressure devices in clinical practice is their measurement accuracy and reproducibility of results. Standardized protocols for validating the clinical accuracy of non-invasive blood pressure measuring devices have been available since 1987, some of which were developed by standards bodies and others by professional organizations^*^ (1).

In 2018, experts from the European Society of Hypertension, the Association for the Advancement of Medical Instrumentation and ISO published an endorsement of the ISO 81060-2; 2018 protocol, calling it the “single universal standard” and stating that it “will replace all other previous standards/protocols” ^**^ (1, 88).

Moreover, ISO 81060-3, “Non-invasive sphygmomanometers – Part 3: Clinical investigation of continuous automated measurement type” has been published in December 2022 (89).

Analysis of the patent landscape of non-invasive continuous blood pressure monitoring cuff-based medical sensors suggests that the devices CNAP (CNSystems, Austria), ClearSight (Edwards Lifesciences, United States), LiDCO Hemo-dynamic Monitor (Lidco Group Plc, England), Finapres/Finometer (Finapres Medical Systems, Netherlands), and VitalStream (Caretaker Medical, United States) are FDA-approved and commercialized on the market. These cuff-based devices are certified according to ISO 81060-2, ISO 81060-3, ISO /TS 81060–5 standards (FDA website https://www.accessdata.fda.gov/scripts/cdrh/cfdocs/cfRL/rl.cfm). This indicates the measurement accuracy of these devices.

Clinical studies of the above examples of devices confirm in most cases the effectiveness of their use, the accuracy of blood pressure measurements, and their advantages, disadvantages, and limitations when used in intensive care practice. The advantage of the devices is the detection of early postoperative hypotension in real time and the ability, in particular, to integrate with hospital monitoring and accounting systems (the VitalStream system). The disadvantages of these devices include limiting the movement of the hand and the movement of patients when using them, as well as causing discomfort in patients by their prolonged presence under the continuous observation of the cuff on the finger.

To overcome the limitations of the method of Penaz (venous congestion, limitations of use in patients with vascular diseases, and finger pathology), other methods of non-invasive blood pressure monitoring are used in cuffless devices. Generalized features of the clinical use of cuffless devices are given below.

According to the literature, the T-Line TL-200 system and the Aktiia bracelet allow for additional continuous blood pressure monitoring in intensive care. Nevertheless, the T-Line system’s use in patients with multiple organ dysfunction syndrome or morbid obesity is limited. Furthermore, when the hand moves or a few seconds pass after the cessation of any physical activity, effective measurement with the Aktiia bracelet is not provided.

There is evidence to support the possibility of accurate blood pressure monitoring by the Biobeat device during childbirth and heart surgery. The advantages of using the Biobeat device should be noted, in particular, the independence of the measurement results from the body mass index or skin color and the use of a cloud platform.

The use of the ViSi Mobile device reduced the number of patient transfers (by 25%) to intensive care. With the ViSi Mobile device, there was also a decrease in unplanned mortality. However, this difference regarding mortality was statistically unreliable.

Validation of Somnotouch-NIBP according to the ESH (European Society for Hypertension) protocols has been successfully completed, and FDA approval is pending. Data from BPro system studies revealed that caution should be exercised in patients with confirmed preeclampsia. It should be noted that more research is needed on Senti-Cor-100 device to monitor blood pressure during intensive therapy and resuscitation.

There are a number of patented cuffless devices under development and research that have shown their advantages: the possibility of using them not only in adults but in children, including newborns, on the arm or leg (Boppli); the use of seismocardiography (SeismoWatch).

The characteristics of cuffless devices for non-invasive continuous monitoring of blood pressure are listed in Table 2. The analysis revealed that Tensys Medical, United States; Biobeat, Israel; Sotera Wireless, United States; and HealthStats, Singapore are among the companies whose cufless devices for non-invasive continuous monitoring of blood pressure are certified according to ISO 81060-2, ISO 81060-3, ISO/TS 81060-5 standards and FDA approved. This indicates the measurement accuracy of the devices from these companies. The disadvantages of these devices include the need for calibration and charging devices. The patent analysis also revealed that the trends in the creation of cuffless devices for non-invasive continuous blood pressure monitoring are to increase their convenience of use and compact structure (Aktiia bracelet, Boppli device for newborns, SeismoWatch bracelet, Biobeat chest patch); and to achieve lack of influence of body mass index or skin color is established (Biobeat). A number of cuffless devices for non-invasive continuous monitoring of blood pressure are in the research and development stage. Devices that focus on photoplethysmography, analysis of pulse wave velocity, and pulse transit time are awaiting ISO certification.

**Table 2:** The characteristics of cuffless devices for non-invasive continuous monitoring of blood pressure.

Device, company name, country	Measurement technology, device location	Disadvantages	Validation^*^	Status of the device
T-Line system, Tensys Medical, United States	Applanation tonometry, arm	The need for calibration; charging the device	ISO 81060-2, ISO 81060-3, ISO/TS 81060-5	Approved FDA and commercialized
Boppli, PyrAmes, United States	Capacitance, arm or leg	In newborns prone to pressure sores due to the pressure of medical devices, it is recommended not to leave the cuffs in place for a long time.	Clinical data are limited	R&D
Aktiia bracelet, Aktiia SA, Switzerland	Photoplethysmography, arm	The need for calibration; charging the device	ISO81060-2	The device is still awaiting FDA clearance
Biobeat, Biobeat, Israel	Photoplethysmography, arm, thorax	The need for calibration; charging the device; the skin patch can only be used once, although the sensor is intended for multiple uses.	ISO 81060-2, ISO 81060-3, ISO/TS 81060-5	Approved FDA and commercialized
ViSi Mobile, Sotera Wireless, United States	Pulse wave velocity; Pulse transit time; arm	Charging the device	ISO 81060-2, ISO 81060-3, ISO/TS 81060-5	Approved FDA and commercialized
Somnotouch NIBP, Somnomedics GmbH, Germany	Pulse wave velocity; Pulse transit time; arm	Charging the device	It is recommended to use ISO guidance when validating cuffless BP devices.	SOMNOtouch NIBP have undergone validation according to European Society of Hypertension protocols
BPro, Health-Stats, Singapore	Pulse wave analysis, arm	The need for calibration; charging the device	ISO 81060-2, ISO 81060-3, ISO/TS 81060-5	Approved FDA and commercialized
Senti-Cor-100, Sensifree (United States)	Photoplethysmography, finger	The need for calibration; charging the device	Certificate CE	Commercialized
SeismoWatch, Georgia Tech Research Institute and Northwestern University, United States	Photoplethysmography and seismocardiography; arm, thorax	The need for calibration; charging the device	Clinical data are limited	R&D

^*^https://www.accessdata.fda.gov/scripts/cdrh/cfdocs/cfRL/rl.cfm. ^**^ISO 81060-2 Third edition 2018-11 Non-invasive sphygmomanometers – Part 2: Clinical investigation of intermittent automated measurement type [Including: Amendment 1 (2020)]; ^***^ISO 81060-3 First edition 2022-12 Non-invasive sphygmomanometers – Part 3: Clinical investigation of continuous automated measurement type; ^****^ISO/TS 81060-5 First edition 2020-02 Non-invasive sphygmomanometers – Part 5: Requirements for the repeatability and reproducibility of NIBP simulators for testing of automated non-invasive sphygmomanometers.

An analysis of the clinical trials showed that a number of non-invasive medical sensor technologies (cuff-based and cuffless) are quite accurate and comparable to the “gold standard” continuous invasive blood pressure monitoring, and are approved by the FDA for medical application and certified according to ISO 81060-2, ISO 81060-3, ISO/TS 81060-5. Their advantages include the ability to detect hemodynamic disorders quickly, allowing for timely intervention and treatment, as well as improved monitoring capabilities in a variety of clinical settings without the complications associated with invasive devices. In addition, it should be noted that it is possible to use them as additional tools along with standard monitoring in children to improve perioperative safety, as well as in children in critical condition when an invasive arterial catheter cannot be used due to the risk of complications and side effects. It should be emphasized that unregistered and uncertified medical sensors require further clinical trials.

^*^ – Association for the Advancement of Medical Instrumentation (United States), 1987, 1992, and 2002; British Hypertension Society, 1990, 1993; German Hypertension League (Deutsche Hochdruckliga), 1999; European Society of Hypertension, 2002, 2010; ISO, 2009; American National Standards Institute, Association for the Advancement of Medical Instrumentation and International Organization for Standardization, 2009, 2013; Association for the Advancement of Medical Instrumentation, European Society of Hypertension and International Organization for Standardization, 2018.

^**^ – According to ISO 81060-2 for non-invasive sphygmomanometers, the mean overall difference is ±5 mm Hg and the standard deviation is within 8 mm Hg. The mean difference among patients is based on the mean difference in blood pressure and the standard deviation.

## Conclusion

The present review of patent and clinical data has established the effectiveness of a number of non-invasive medical sensors for continuous blood pressure monitoring (cuff-based and cuffless), their accuracy, and their comparability to the “gold standard” of continuous invasive blood pressure monitoring. However, there were also disadvantages and limitations to the use of some devices (as detailed in the discussion section).

Currently, non-invasive medical sensors for continuous blood pressure monitoring do not completely replace but complement existing methods of regular blood pressure measurement, and in the long term, it is hoped that these devices will be further refined and will shape the future of blood pressure monitoring in clinical settings.

## Author contributions

OL, OK, AA, and HW: conceptualization. OL: formal analysis and writing—original draft preparation. OL, AB, EP, JN, MK-P, OK, AA, and HW: writing—review and editing. All authors contributed to the article and approved the submitted version.

## Funding

This study was supported by a fund from the Ludwig Boltzmann Gesellschaft (OL was a fellowship recipient).

## Conflict of interest

The authors declare that the research was conducted in the absence of any commercial or financial relationships that could be construed as a potential conflict of interest.

## Publisher’s note

All claims expressed in this article are solely those of the authors and do not necessarily represent those of their affiliated organizations, or those of the publisher, the editors and the reviewers. Any product that may be evaluated in this article, or claim that may be made by its manufacturer, is not guaranteed or endorsed by the publisher.

## Footnotes

^1^ https://www.globenewswire.com/

^2^ https://www.accessdata.fda.gov/scripts/cdrh/cfdocs/cfRL/rl.cfm
