# Supplementary material for: Patent landscape review of non-invasive medical sensors for continuous monitoring of blood pressure and their validation in critical care practice
Source: Front Med (Lausanne). 2023 Jul 11;10:1138051. doi: 10.3389/fmed.2023.1138051 (PMC10366595; doi:10.3389/fmed.2023.1138051)
Supplement: Supplementary file 1 [file Data_Sheet_1.docx]

Supplementary Information

Patent landscape review of non-invasive medical sensors for continuous monitoring of blood pressure and their validation in critical care practice

Olena Litvinova^1*^, Aylin Bilir^2^, Emil D. Parvanov^2,3^, Josef Niebauer ^4,5,6^, Maria Kletecka-Pulker^2,7^, Oliver Kimberger^2,8^, Atanas G. Atanasov^2,9*^, and Harald Willschke^2,8*^

^1^National University of Pharmacy of the Ministry of Health of Ukraine, Kharkiv, Ukraine

^2^Ludwig Boltzmann Institute Digital Health and Patient Safety, Medical University of Vienna,

Vienna, Austria

^3^Department of Translational Stem Cell Biology, Research Institute of the Medical University of Varna, Varna, Bulgaria

^4^ Ludwig Boltzmann Institute for Digital Health and Prevention, Salzburg, Austria

^5^ University Institute of Sports Medicine, Prevention and Rehabilitation, Paracelsus Medical University Salzburg, Salzburg, Austria

^6^ REHA Zentrum Salzburg, Salzburg, Austria

^7^Institute for Ethics and Law in Medicine, University of Vienna, Vienna, Austria

^8^Department of Anaesthesia, Intensive Care Medicine and Pain Medicine, Medical University of Vienna, Vienna, Austria

^9^Institute of Genetics and Animal Biotechnology of the Polish Academy of Sciences, Jastrzebiec, Poland

*** Correspondence:**Olena Litvinova

[hlitvinova@gmail.com](mailto:hlitvinova@gmail.com)

Atanas G. Atanasov

[Atanas.Atanasov@dhps.lbg.ac.at](mailto:Atanas.Atanasov@dhps.lbg.ac.at)

Harald Willschke

[harald.willschke@meduniwien.ac.at](mailto:harald.willschke@meduniwien.ac.at)

**Supplementary Table S1.** The International Patent Classification and Cooperative Patent Classification codes are used in the patent search.

| **Code** | **Meaning** |
| --- | --- |
| ***International Patent Classification*** | |
| **A61B5/00** | **Measuring for diagnostic purposes** |
| **A61B5/02** | **Measuring pulse, heart rate, blood pressure or blood flow** |
| A61B5/021 | Measuring pressure in heart or blood vessels |
| A61B5/022 | by applying pressure to close blood vessels (e.g. against the skin) |
| A61B5/0225 | the pressure being controlled by electric signals |
| A61B5/024 | Measuring pulse rate or heart rate |
| A61B5/0245 | using sensing means generating electric signals |
| A61B5/0255 | Recording instruments specially adapted therefor |
| A61B5/026 | Measuring blood flow |
| A61B5/0265 | using electromagnetic means, e.g. electromagnetic flow meter |
| A61B5/0285 | Measuring phase velocity of blood waves |
| A61B5/029 | Measuring blood output from the heart |
| A61B5/0295 | using plethysmography |
| **A61B5/05** | **Detecting, measuring or recording for diagnosis by means of electric currents or magnetic fields; Measuring using microwaves or radio waves** |
| A61B5/053 | Measuring electrical impedance or conductance of a portion of the body |
| A61B5/1455 | using optical sensors |
| **A61B8/00** | **Diagnosis using ultrasonic, sonic or infrasonic waves** |
| A61B8/02 | Measuring pulse or heart rate |
| A61B8/04 | Measuring blood pressure |
| A61B8/06 | Measuring blood flow |
| **G06F17/00** | **Digital computing or data processing equipment or methods, specially adapted for specific functions** |
| **G06F19/00** | **Digital computing or data processing equipment or methods, specially adapted for specific applications** |
| **G16H40/67** | **Information and communication technology (ICT) specially adapted for the management or administration of healthcare resources or facilities; ICT specially adapted for the management or operation of medical equipment or devices for remote operation** |
| ***Cooperative Patent Classification*** | |
| **A61B2560/0223** | **of calibration, e.g. protocols for calibrating sensors** |
| A61B2562/0247 | Pressure sensors |
| A61B2562/04 | Arrangements of multiple sensors of the same type |
| **A61B5/0002** | **Remote monitoring of patients using telemetry** |
| A61B5/0022 | Monitoring a patient using a global network, e.g. telephone networks, internet |
| A61B5/0024 | for multiple sensor units attached to the patient, e.g. using a body or personal area network |
| A61B5/0059 | using light |
| **A61B5/02** | **Detecting, measuring or recording pulse, heart rate, blood pressure or blood flow** |
| **A61B5/021** | **Measuring pressure in heart or blood vessels** |
| A61B5/02108 | from analysis of pulse wave characteristics |
| A61B5/02116 | of pulse wave amplitude |
| A61B5/02125 | of pulse wave propagation time |
| A61B5/02133 | by using induced vibration of the blood vessel |
| **A61B5/022** | **by applying pressure to close blood vessels (e.g. against the skin)** |
| A61B5/02241 | of small dimensions, e.g. adapted to fingers |
| A61B5/0225 | the pressure being controlled by electric signals |
| A61B5/02255 | the pressure being controlled by plethysmographic signals, e.g. derived from optical sensors |
| **A61B5/024** | **Detecting, measuring or recording pulse rate or heart rate take precedence** |
| A61B5/02416 | using photoplethysmograph signals (e.g. generated by infra-red radiation) |
| A61B5/02427 | Details of sensor |
| A61B5/02438 | with portable devices, e.g. worn by the patient |
| **A61B5/026** | **Measuring blood flow take precedence** |
| A61B5/0261 | using optical means |
| **A61B5/0285** | **Measuring or recording phase velocity of blood waves** |
| **A61B5/029** | **Measuring or recording blood output from the heart, e.g. minute volume takes precedence** |
| A61B5/0295 | using plethysmography |
| **A61B5/6801** | **specially adapted to be attached to or worn on the body surface** |
| A61B5/681 | Wristwatch-type devices |
| A61B5/6824 | Arm or wrist |
| A61B5/6826 | Finger |
| A61B5/683 | Means for maintaining contact with the body |
| A61B5/6831 | Straps, bands or harnesses |
| A61B5/6838 | Clamps or clips |
| A61B5/6843 | Monitoring or controlling sensor contact pressure |
| **A61B5/72** | **Signal processing specially adapted for physiological signals or for diagnostic purposes** |
| A61B5/7203 | for noise prevention, reduction or removal |
| A61B5/7207 | of noise induced by motion artifacts |
| A61B5/721 | using a separate sensor to detect motion or using motion information derived from signals other than the physiological signal to be measured |
| A61B5/7221 | Determining signal validity, reliability or quality take precedence |
| A61B5/7239 | using differentiation including higher order derivatives |
| A61B5/742 | using visual displays |
| A61B5/743 | Displaying an image simultaneously with additional graphical information |
| **A61B8/04** | **Measuring blood pressure** |
| A61B8/06 | Measuring blood flow |
| **G06F1/163** | **Electric digital data processing. Wearable computers (e.g. on a belt)** |
| **G16H10/40** | **Healthcare informatics for data related to laboratory analysis** |
| **G16H40/67** | **healthcare informatics for remote operation** |
| **G16H50/20** | **healthcare informatics for computer-aided diagnosis, e.g. based on medical expert systems** |
| **G16H50/30** | **healthcare informatics for calculating health indices for individual health risk assessment** |

**Supplementary Figure S1.** A flow diagram for non-invasive blood pressure monitoring by the ClearSight (Nexfin). Diagram modified from reference [Andrew B., Ernest H. (2020). Finger cuff device with non-volume clamp plethsmography method for continuous non-invasive blood pressure measurement (WO 2020/176207 A1)].

**Supplementary Figure S2.** The process of maintaining the optimal applanation level of the T-Line system. Diagram modified from reference [Finburgh SE, Katayama AS, Vidischak R J, Butler AT, Kurt B. (2021). Apparatus and methods for non-invasively measuring a patient’s arterial blood pressure (US 10952675 B2)].

**Supplementary Figure S3.** A flow chart for measuring cNIBP, SpO2, respiration rate, heart rate, temperature, and motion according to the ViSi Mobile. Diagram modified from reference [Devin M., Guanqun Z., Isaac H. (2016). Body-worn system for continuous, noninvasive measurement of vital signs (EP 3102097 A1)].
